# Near-natural transformation of *Pinus tabuliformis* better improve soil nutrients and soil microbial community

**DOI:** 10.7717/peerj.12098

**Published:** 2021-09-23

**Authors:** You Yin, Qiuli Li, Haitao Du

**Affiliations:** 1 College of Forestry, Shenyang Agricultural University, Shenyang, Liaoning, China.; 2 Research Station of Liaohe-River Plain Forest Ecosystem, Chinese Forest Ecosystem Research Network (CFERN), Shenyang Agricultural University, Tieling, Liaoning, China

**Keywords:** *Pinus tabuliformis*, Near-natural transformation, Soil characteroistics, Soil microbial community

## Abstract

*Pinus tabulaeformis* plantations have been established around northern China to restore degraded land and provide timber or fuelwood. In recent years, widely distributed monoculture *P. tabulaeformis* forests have been transformed into mixed forests due to various ecological problems. However, the current research on the influence of near-natural transformation of *P. tabulaeformis* on soil microbial diversity and community composition remains limited. Therefore, we examined the effect of forest conversion from monoculture* Pinus tabuliformis* (PT) to *P. tabuliformis*-*Armeniaca vulgaris* (PTAU), *P. tabuliformis* - *Robinia pseudoacacia* (PTRP), P. *tabuliformis* - *Vitex negundo* L. var. *heterophylla* (PTVN) forests on soil microbial community diversity and composition. The results indicated that compared to PT, PTAU, PTVN, and PTRP could enhance the soil pH, TC, TN, AN, and AK in different degrees, the most obvious in PTAU. Near-natural transformation of *P. tabuliformis* could improve soil bacterial Pielou_e index, and Simpson index, as well as soil fungal Chao1 index. Proteobacteria and Ascomycota were the dominant soil microbial community at the phylum level. What’s more, both soil bacterial and fungal community among PT, PTAU, PTRP and PTVN showed clear different, and PTAU obviously altered the soil microbial community structure. Proteobacteria was the predominant group in PT, while, Gemmatimonadetes enriched in PTVN. Ascomycota was the predominant group in PTAU, while, Basidiomycota was the predominant group in PTRP. Near-natural transformation of *P. tabuliformis* could change soil microbial community via altering soil characteristics. In brief, our research results revealed the influence of tree composition and soil nutrient availability on soil microbial diversity and composition, and provided management guidance for introduction soil microbial community in forest protection and management.

## Introduction

Desertification has always been a global important ecological environmental problem in the arid and semi-arid regions (Becerril-Piña et al., 2015), which has caused a loss of soil nutrients, a decline in land productivity and environmental degradation (Li et al., 2018). In order to solve the current ecological problems, China has implemented large-scale national projects for decades, mainly through afforestation and reforestation to restore degraded land and improve ecological environment (Xu et al., 2013). Currently, 31.8% of forest area in China is plantations (State Forest Administration, 2010), especially coniferous monoculture forests (Sheng, 2018), which are increasingly becoming an important part of the world’s forest (Keenan et al., 2015). *Pinus tabuliformis* is an endemic tree species with wide distribution and strong adaptability to large areas in the northern China (Shi et al., 2013), which is also the zonal vegetation in the humid and sub-humid climate zone of warm temperate zone. *P. tabuliformis* plantation forest has played a momentous and positive role in adjusting the climate, controlling soil erosion, changing the local natural appearance, and promoting the development of agriculture and animal husbandry (Wang et al., 2019). However, there is growing evidence suggesting that pure *P. tabulaeformis* forest have ineluctable disadvantages with low biodiversity of understory, severe growth decline, poor resistance to natural disasters, soil degradation, and forest degradation (Shi et al., 2013; Sheng, 2018), which seriously hinders the healthy development of *P. tabulaeformis* plantation. Therefore, the ecological problems of plantations have received greater attention from scientists and forest managers.

It is gradually realized that the conversion of pure *P. tabulaeformis* forests is the pivotal problem that needs to be resolved in the development of current plantation (Li et al., 2021a). At present, in order to overcome these disadvantages and improve the plantation forest ecosystem functions, many regions of the world have carried out “near-natural transformation” (Lu, 2006), including selective logging (Luo et al., 2013), creating gap (Rouvinen & Kouki, 2011), establishment of broad-leaved species in mixtures with conifers (Wang & Liu, 2011; Nakamura, Morimoto & Mizutani, 2005). And creating a suitable mixed broadleaf-conifer forest ecosystem is one of the efficient measures to develop biodiversity, maintain forest land productivity, increase the nutrient turnover rate (Li, Yang & Wei, 2016), improve the forest stability and resistance, as well as promote effectiveness sustainability of plantations (Dănescu, Albrecht & Bauhus, 2016). The near-natural transformations of *Cryptomeria japonica* (Taki et al., 2010), *P. massoniana* (Luo et al., 2013), *P. tabuliformis* (Wang & Liu, 2011), and *Cunninghamia lanceolata* (He et al., 2013) plantations have been well documented.

Soil physical and chemical properties, as important indicators of soil quality, have an important impact on soil water storage performance and absorption, soil nutrients utilization and transformation, thereby directly or indirectly affecting the growth and development of forest trees and forest land productivity. Soil microorganism represent a major proportion of terrestrial biodiversity and play an essential role in many ecological processes (Cotrufo et al., 2015), such as soil organic matter decomposition and nutrients cycling (Chen et al., 2019). Furthermore, as the soil microbial community diversity increases, the soil sustainable use and its resistance to soil-borne disease increase (Kaschuk, Alberton & Hungria, 2011). Compared to soil characteristics, soil microorganisms are more sensitive to the transformation of the forest. Under the similar climatic conditions and local site conditions, near-natural transformation of pure forest has been frequently investigated to influence the soil bacterial and fungal community diversity (Liu et al., 2018; Xiao et al., 2019) and composition (Guo et al., 2016). Accumulating evidence suggests that tree species could influence soil microbial communities through litter, root exudates and symbiotic mycorrhizal fungi (Prescott & Grayston, 2013). What’s more, soil characteristics are a strong determinant for soil microbial diversity and composition (Nakayama et al., 2019). Variations of the soil environment caused by the conversion of forest types will also affect microbial diversity and composition (Meng et al., 2020). Despite the importance of soil microbial communities to ecosystem functions (Li et al., 2019), the impact of near-natural forests conversion on soil microbial diversity and composition remain poorly understood.

At present, most of the *P. tabulaeformis* plantations in western Liaoning are 30–50 years old and have entered the mature or near-mature stage. The vitality of the trees began to weaken, and the phenomenon of dieback increased. The problem of renewal need to be solved urgently. After decades of near-natural development, broadleaved tree species, such as *Armeniaca vulgaris*, *Robinia pseudoacacia*, *Vitex negundo* L. var. *heterophylla,* have introduced in the *P. tabuliformis* forest, forming a coniferous-broadleaved mixed forest, including *P. tabuliformis-Armeniaca vulgaris* mixed forest, *P*. *tabuliformis* - *Robinia pseudoacacia* mixed forest, *P*. *tabuliformis*-*Vitex negundo* L. var. *heterophylla* mixed forest. In present study, we tested the effects of near-natural conversion of *P. tabuliformis* on soil nutrients and the soil microbial community. Specifically, we hypothesized that (1) soil nutrients in the near-natural forests would be higher than in the *P. tabuliformis* plantations; (2) soil microbial community diversity in the near-natural forests would be higher than in the plantations, and soil microbial community composition existed difference among different vegetation types; (3) changes in the soil microbial community diversity and composition were correlated with the soil characteristics induced by forest type conversion. These results will help identify the difference of soil properties and microbial community among different forest types, which will provide scientific basis and theoretical suggestions regarding the sustainable plantation forest management.

## Material and Method

### Research area

The study area was carried out at the Shahai experimental area (41°29′N, 119°27′E), which is located in the Chaoyang, Liaoning Province. The climate belongs to semi-arid monsoon climate zone, with an annual mean temperature of 7−10 °C, with an absolute maximum temperature of 40.7 °C and minimum temperature of −26.4 °C, and a frost-free period of 145–150 days throughout the year. The annual average precipitation is 400–600 mm, with 60%–70% concentrated in June–August, the annual evaporation is about 1,600–1,800 mm, which is 3 to 4 times the precipitation, and the annual average relative humidity is 50%–60%. This area belongs to the edge of North China flora, the transition zone between North China and Mongolia flora. Woody plants include *P. tabulaeformis*, *Robinia pseudoacacia*, *Quercus mongolica*, *Prunus sibirica*, *Prunus dabian*, *Zizyphus jujuba* var. *inermis*, *Hippophae rhamnoides*, *Vitex chinensis*, *Zizyphus jujube* var. *spinosus* and *Ostryopsis davidiana*. The herbaceous plants are mainly *Arundinella hirta*, *Cleistogenes polyphylla* and *Aneurolepidium chinensis*. The typical soils are classified as Eluvial brown soil according to the FAO Taxonomy. Large areas of *P. tabuliformis* forests were planted in the 1950s, the government implemented the Natural Forest Conservation Program (NFCP) over the last 20 years (Zhang et al., 2000). Currently, *P. tabuliformis* (PT) has largely been transformation to *Pinus tabuliformis -Armeniaca vulgaris* (PTAU), *Pinus tabuliformis - Robinia pseudoacacia* (PTRP), and *Pinus tabuliformis - Vitex negundo* L. var. *heterophylla* (PTVN) (Table S1).

### Samples collection

Four forest types were selected in this study in August 2020. In order to reduce the spatial autocorrelation, we established four spatially interspersed stands with 0.06 ha (20 m ×30 m) at the distances >300 m as the repetition in each forest type. After removing the litter layer, we collected the 10–15 soil samples of topsoil (0∼10 cm) using soil auger with an “S” shape and mixed together to form a single composite sample for each triplicate plot, giving a total of 16 samples. All collected soil samples were sealed in plastic bags, and then put into icebox for transport to the laboratory. Soil samples for chemical analysis were initially air-dried, finely ground, and sifted through a 100-mesh (0.15 mm) sieve. The microbial DNA samples were stored at −80 °C for DNA extraction.

### The determination of soil characteristics

The soil pH was assayed *via* a 1:2.5 soil: water (w/v) suspensions using pH meter (Mettler Toledo pH (FE20)). The concentrations of soil total carbon (TC) and total nitrogen (TN) were quantified with an elemental analyzer (Euro Vector EA3000). Soil total phosphorus (TP) and available P (AP) contents were determined following soil digestion with H_2_SO_4_-HClO_4_ and soil extraction with 0.5 mol l^−1^ NaHCO_3_, respectively, and assessed by the spectrophotometer (UV-9000S). The concentration of available N (AN) was determined by the alkali solution diffusion method. The soil C/N ratio was calculated as their mass ratios of respective elements. The determination of available potassium (AK) was performed atomic absorption spectrometry after extraction with 1.0 mol L^−1^ NH_4_OAc.

### Soil DNA extraction and Illumina sequencing

Briefly, genomic DNA was extracted from 0.5 g of soil using the FastDNA SPIN Kit (MP Biomedicals, Santa Ana, CA, USA), in accordance with the instructions provided by the manufacturer. The target bacterial V3-V4 regions of 16S rRNA gene were amplified using the forward primer 338F (5′-ACTCCTACGGGAGGCAGCAG-3′) and reverse primer 806R (5′-GGACTACHVGGGTWTCTAAT-3′). And the target fungal DNA gene ITS1 regions were amplified using the forward primer ITS1F (5′-CTTGGTCATTTAGAGGAAGTAA-3′) and reverse primer ITS2 (5′-GCTGCGTTCTTCATCGATGC-3′). The total PCR reaction were carried out with 25 µl mixture, including 2 µl of dNTPs (2.5 mM), 2µl (40–50 ng) of DNA Template, 8.75 µl of ddH_2_O, 10 uM (1 µl) of forward primer, 10 uM (1 µl) of reverse primer, 5 U/µl (0.25 µl) of Q5 High-Fidelity DNA Polymerase, 5 µl of Q5 High-Fidelity GC buffer (5 ×), and 5 µl of Q5 reaction buffer (5 ×) (Deng et al., 2018). The following PCR thermal cycling condition consisted of an initial denaturation step at 98 °C for 5 min, followed by 25 cycles of denaturation at 98 °C for 15 s, annealing at 55 °C for 30 s, and elongation at 72 °C for 30 s, with final elongation at 72 °C for 5 min. The PCR amplicons were further purified by Agencourt AMPure Beads (Beckman Coulter, Indianapolis, IN) and quantified using PicoGreen dsDNA Assay Kit (Invitrogen, Carlsbad, CA, USA). The purified amplicons of bacterial and fungal genes were paired-end sequenced on Illumina NovaSeq PE250 platform at Shanghai Personal Biotechnology Co., Ltd, Shanghai, China. The high-throughput sequencing raw data of bacteria and fungi were uploaded in the NCBI database with the SRA accession number of PRJNA720506.

### Bioinformation analysis

The obtained raw 16S rRNA and ITS rRNA sequences data were processed (*i.e.,* filtering, clustering, trimming and chimera checking) using the Quantitative Insights Into Microbial Ecology (QIIME) pipeline with default settings. And the operational taxonomic unit (OTUs) were classified at 97% confidence level and clustered by the UPARSE pipeline with a cutoff of occupancy (100%, detected at every location) and relative abundance (>1%). Taxonomic characterization of the representative sequences of bacterial OTUs was performed using the SILVA 16S rRNA database and Unite database.

### Statistical analyses

The significant differences in soil physical and chemical properties, the microbial community diversity index, and soil microbial community composition of different forest types were examined with one-way analysis of variance (ANOVA) followed by the least significant difference (LSD) at the 95% confidence level. The shared and unique OTUs among different samples were calculated and visualized using the package of “VennDiagram” in RStudio (Zaura et al., 2009). Non-metric multidimensional scaling (NMDS) analysis of the phylogenetic of OTUs based on Jaccard distance was used to better visualize and investigate the distribution characteristics and the dissimilarity of soil microbial communities among different samples using RStudio with the package of “Vegan” (Lozupone et al., 2007). Permutational multivariate analysis of variance (permanova) was used to analyze the differences between groups using RStudio with the package of “Vegan”. The correlations between soil properties and soil community diversity were studied using Spearman’s rank correlations using the package of “VennDiagram” in RStudio (R Development Core Team, 2009). LEFSE analysis, namely LDA Effect Size analysis, achieved by the rank sum test of non-parametric factor Kruskal-Wallis, can be used to find the groups with significant differences in abundance among different samples (Segata et al., 2011). In order to explore the contribution of edaphic variables on the soil dominant communities at phylum level, redundancy analysis was carried out using CANOCO for Windows, Version 4.5 (Braak & Smilauer, 2002).

## Results

### Soil characteristics among different vegetation types

Except for AP, soil pH (*F* = 43.38, *P* = 0.00), TC (*F* = 8.15, *P* = 0.00), TN (*F* = 5.72, *P* = 0.01), C/N (*F* = 18.76, *P* = 0.00), TP (*F* = 16.34, *P* = 0.00), AN (*F* = 5.68, *P* = 0.01), and AK (*F* = 4.47, *P* = 0.03) existed significant differences among different vegetation types. Compared to PT, PTAU, PTVN and PTRP could increase the soil pH, TC, TN, AN, and AK in different degrees, the most obvious in PTAU with 7.71, 20.10 g/kg, 1.48 g/kg, 108.38 mg/kg, and 102.80 mg/kg. Soil C/N showed the highest in PT with 14.76, followed by PTAU, PTVN and PTRP (Table 1).

**Table 1:** Soil characteristics under four different vegetation types.

	PTAU	PTRP	PT	PTVN	df	F	P
pH	7.71 ± 0.07aA	7.64 ± 0.20aA	6.75 ± 0.17cC	7.20 ± 0.04bB	3	43.38	0.00
TC(g/kg)	20.10 ± 2.65aA	14.80 ± 1.78bB	13.17 ± 1.74bB	13.56 ± 2.64bB	3	8.152	0.00
TN(g/kg)	1.48 ± 0.37aA	1.45 ± 0.09aA	0.90 ± 0.14bB	1.26 ± 0.20aAB	3	5.722	0.01
C/N	13.92 ± 1.80aA	10.18 ± 0.87bB	14.76 ± 0.35aA	10.77 ± 0.52bB	3	18.76	0.00
TP(g/kg)	0.41 ± 0.08aAB	0.48 ± 0.03aA	0.33 ± 0.03bBC	0.26 ± 0.02bC	3	16.34	0.00
AN(mg/kg)	108.38 ± 25.08aA	101.80 ± 10.21a	62.38 ± 12.95bB	95.20 ± 16.56aAB	3	5.68	0.01
AP(mg/kg)	3.60 ± 0.83aA	3.23 ± 0.46aA	3.38 ± 0.46aA	2.93 ± 0.47aA	3	0.96	0.44
AK(mg/kg)	102.80 ± 22.85a	100.20 ± 20.23aAB	65.23 ± 14.06bB	79.18 ± 4.00abAB	3	4.47	0.03

**Notes.**

Data are means ± standard deviation (*n* = 4).

TC total carbon TN total nitrogen TP total phosphorus AN available nitrogen AP available phosphorus AK available potassium PT
*Pinus tabuliformis*
 PTAU *Pinus tabuliformis*-*Armeniaca vulgaris* mixed forest PTRP *Pinus tabuliformis*-*Robinia pseudoacacia* mixed forest PTVN *Pinus tabuliformis*-*Vitex negundo* L. var. *heterophylla* mixed forest

Different uppercase letters in the same row indicate significant difference at the 0.01 level, and different lowercase letters in the same row indicate significant differences at the 0.05 level.

### Soil microbial community among different vegetation types

**Figure 1:**
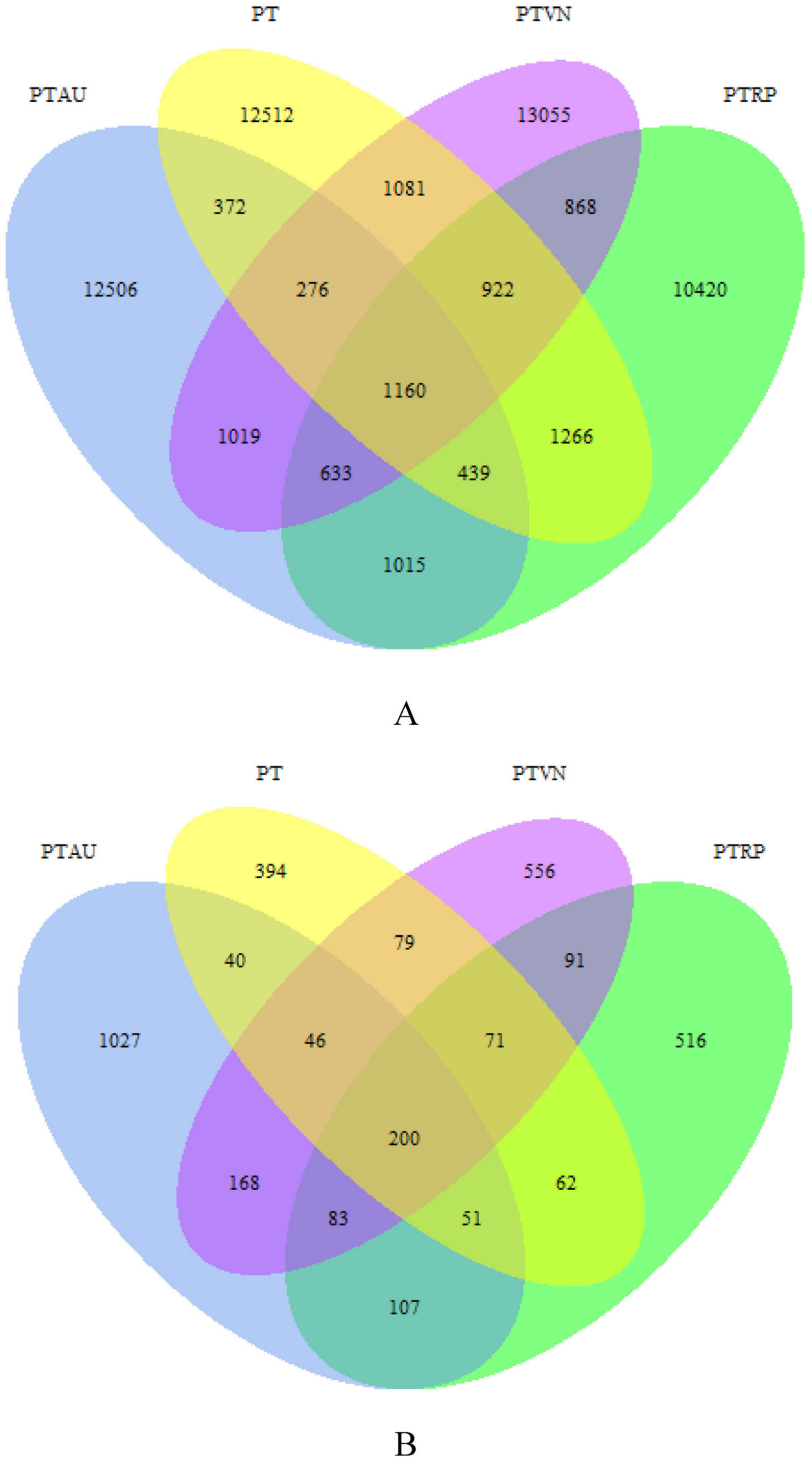
The unique and shared OTUs of soil bacteria (A) and fungi (B). PT, *Pinus tabuliformis*; PTAU, *Pinus tabuliformis*-*Armeniaca vulgaris* mixed forest; PTRP, *Pinus tabuliformis*-*Robinia pseudoacacia* mixed forest; PTVN, *Pinus tabuliformis*-*Vitex negundo* L. var. *heterophylla* mixed forest.

A total of 1,097,939 high quality bacterial sequences were obtained across all samples, with average 68,621 sequences per sample, which were clustered into 57,544 OTUs. The number of shared OTUs among PT, PTAU, PTVN and PTRP was 1,160, and the unique OTUs was 12,512, 12,506, 13,055, 10,420 in PT, PTAU, PTVN, PTRP, accounting for 69.40%, 71.79%, 68.66%, and 62.31% of their total OTUs, respectively (Fig. 1A). A total of 2,302,306 high quality fungal sequences were obtained across all samples, with average 115,115 sequences per sample, which were clustered into 3,492 OTUs. The number of shared OTUs among PT, PTAU, PTVN, and PTRP was 200, and the unique OTUs was 394, 1,027, 556, 516 in PT, PTAU, PTVN, and PTRP, accounting for 41.78%, 59.64%, 42.97%, and 43.69% of their total OTUs, respectively (Fig. 1B).

There was considerable variation of soil bacterial Pielou_e index (*F* = 13.00, *P* = 0.00), and Simpson index (*F* = 9.52, *P* = 0.00) among PT, PTAU, PTRP, and PTVN. Soil bacterial Pielou_e index and Simpson index appeared the highest in PTVN with 0.905 and 0.999, respectively, followed by PTRP and PTAU, and lowest in PT with 0.881 and 0.998. However, soil bacterial Shannon index (*F* = 2.35, *P* = 0.12) and Chao1 index (*F* = 0.55, *P* = 0.66) appeared no significant differences among different samples (Table 2). Furthermore, soil fungal Pielou_e index (*F* = 2.31, *P* = 0.13), Shannon index (*F* = 3.03, *P* = 0.07), and Simpson index (*F* = 1.01, *P* = 0.42) also existed no obvious difference among PT, PTAU, PTRP and PTVN. While, soil fungal Chao1 index was the highest in PTAU, followed by PTVN and PTRP, and lowest in PT with 409.33 (Table 2).

**Table 2:** Soil community diversity among four different vegetation types.

**Bacteria**	**Chao1 index**	**Pielou_e index**	**Shannon index**	**Simpson index**
PTAU	6138.70 ± 767.75aA	0.900 ± 0.005aA	11.22 ± 0.16abA	0.9988 ± 0.0002aAB
PTRP	6458.93 ± 966.47aA	0.911 ± 0.004aA	11.38 ± 0.14abA	0.9991 ± 0.0000aA
PT	6640.46 ± 855.57aA	0.881 ± 0.013bB	11.05 ± 0.30bA	0.9983 ± 0.0004bB
PTVN	7077.26 ± 1480.11aA	0.905 ± 0.003aA	11.43 ± 0.24aA	0.9991 ± 0.0001aA
df	3	3	3	3
F	0.55	13.00	2.35	9.52
P	0.66	0.00	0.12	0.00
**Fungi**	**Chao1 index**	**Pielou_e index**	**Shannon index**	**Simpson index**
PTAU	666.80 ± 46.21aA	0.78 ± 0.02aA	7.32 ± 0.20aA	0.98 ± 0.00aA
PTRP	462.95 ± 134.69bcB	0.55 ± 0.25bA	4.96 ± 2.32bA	0.79 ± 0.32aA
PT	409.33 ± 32.59cB	0.60 ± 0.09abA	5.23 ± 0.80bA	0.90 ± 0.08aA
PTVN	544.71 ± 46.07bAB	0.69 ± 0.02abA	6.25 ± 0.19abA	0.96 ± 0.01aA
df	3	3	3	3
F	8.56	2.31	3.03	1.01
P	0.00	0.13	0.07	0.42

**Notes.**

Data are means ± standard deviation (*n* = 4).

TC total carbon TN total nitrogen TP total phosphorus AN available nitrogen AP available phosphorus AK available potassium PT
*Pinus tabuliformis*
 PTAU *Pinus tabuliformis*-*Armeniaca vulgaris* mixed forest PTRP *Pinus tabuliformis*-*Robinia pseudoacacia* mixed forest PTVN *Pinus tabuliformis*-*Vitex negundo* L. var. *heterophylla* mixed forest

Different uppercase letters in the same row indicate significant difference at the 0.01 level, and different lowercase letters in the same row indicate significant differences at the 0.05 level.

Soil bacterial Pielou_e index existed obviously positive relation with soil pH (*r* = 0.52, *P* = 0.05) and TN (*r* = 0.67, *P* = 0.01). Soil C/N showed dramatically negative correlation with Pielou_e index (r = −0.76, *P* = 0.01), Shannon index (r = −0.68, *P* = 0.01), and Simpson index (r = −0.92, *P* = 0.01) (Table 3). Nevertheless, no significant correlations of soil fungal community diversity with soil characteristics were observed (Table 3).

### Soil microbial community composition among different vegetation types

**Figure 2:**
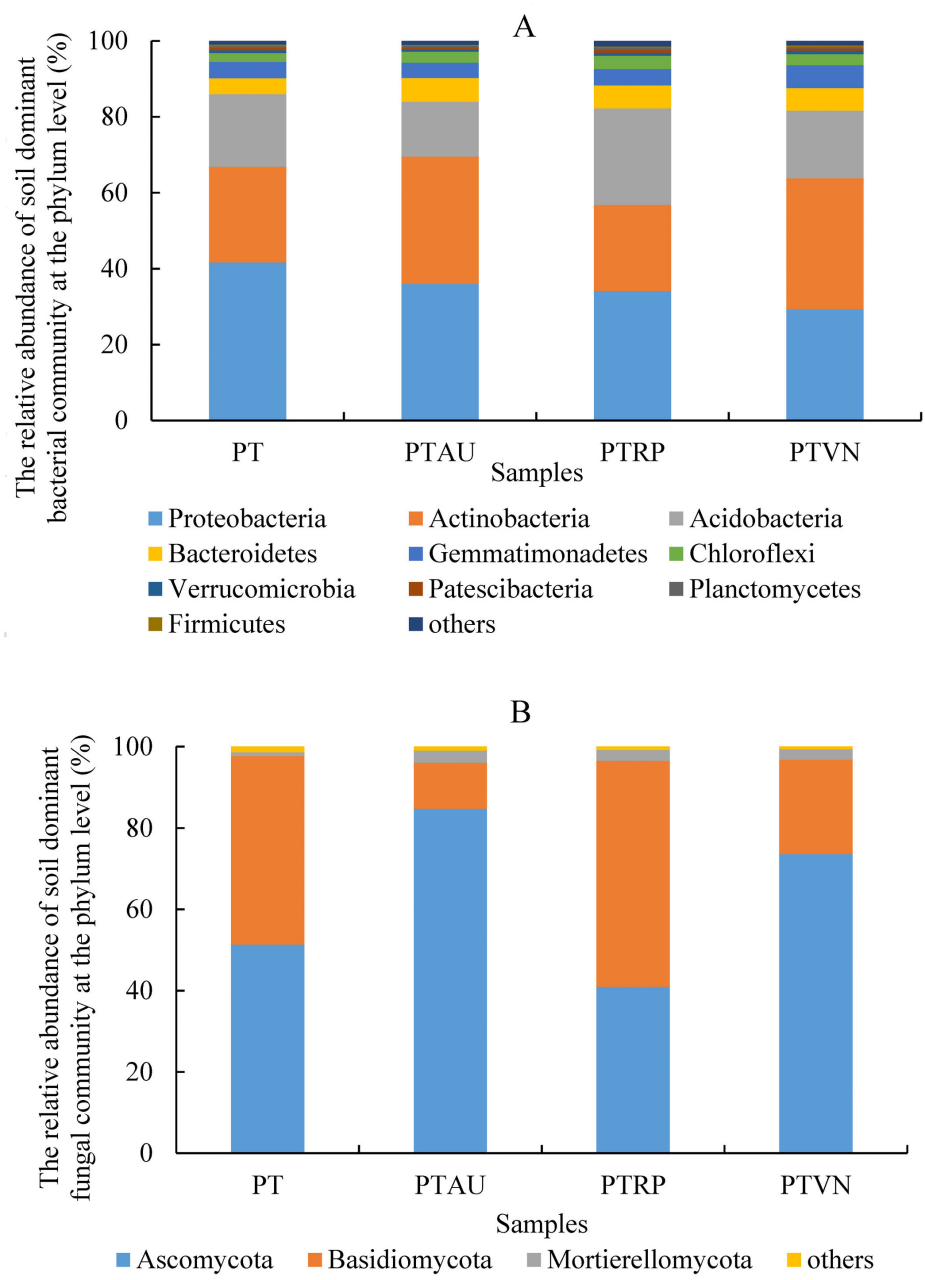
Soil bacterial (A) and fungal (B) community ranking at the top ten at the phylum level with the relative abundance more than 1%. PT, *Pinus tabuliformis*; PTAU, *Pinus tabuliformis*-*Armeniaca vulgaris* mixed forest; PTRP, *Pinus tabuliformis*-*Robinia pseudoacacia* mixed forest; PTVN, *Pinus tabuliformis*-*Vitex negundo* L. var. *heterophylla* mixed forest.

**Figure 3:**
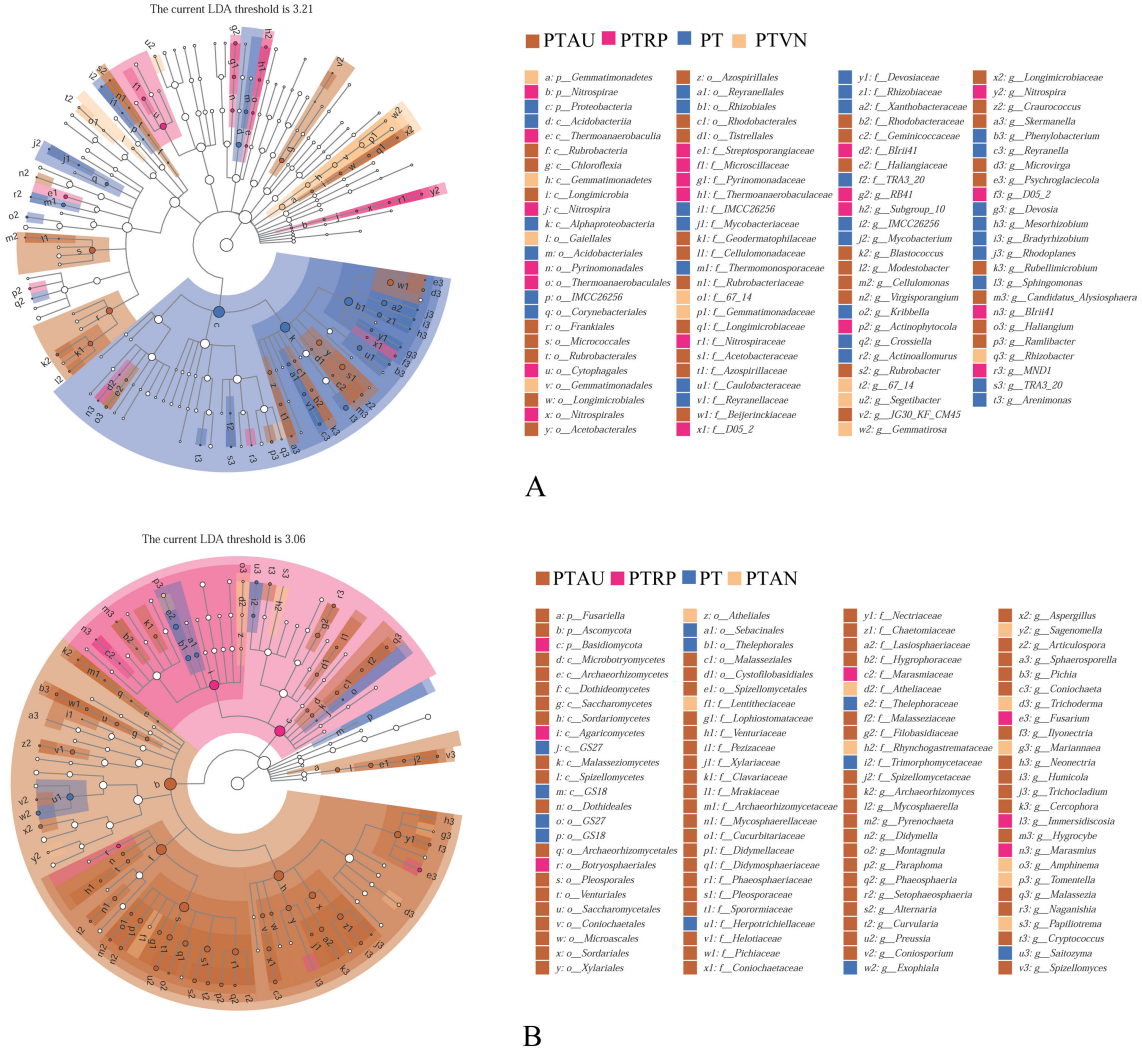
Lefse of soil bacterial community (A) and fungal community (B) under different vegetation types. PT, *Pinus tabuliformis*; PTAU, *Pinus tabuliformis*-*Armeniaca vulgaris* mixed forest; PTRP, *Pinus tabuliformis*-*Robinia pseudoacacia* mixed forest; PTVN, *Pinus tabuliformis*-*Vitex negundo* L. var. *heterophylla* mixed forest.

Soil bacterial communities ranking at the top ten were Proteobacteria, Actinobacteria, Acidobacteria, Bacteroidetes, Gemmatimonadetes, Chloroflexi, Verrucomicrobia, Patescibacteria, Planctomycetes, and Firmicutes, accounting for 99.05%, 99.01%, 98.54% and 98.83% in PT, PTAU, PTRP, and PTVN, respectively (Fig. 2A). The relative abundance of Proteobacteria in PT was the highest with 41.67%, followed by PTAU, PTTRP, and lowest in PTVN. While, Actinobacteria and Gemmatimonadetes in PTVN appeared the highest with 34.48%, and 6.07%. What’s more, lefse with LDA of 3.21 indicated that Proteobacteria were the predominant group in PT, while, Gemmatimonadetes enriched in PTVN at the phylum level, (Fig. 3A). At the genus level, the average relative abundance of soil bacterial community more than 1% were *Subgroup_6*, *RB41*, *Sphingomonas*, *Blastococcus*, *Pseudonocardia*, *Solirubrobacter*, *Gemmatimonas*, *67-14*, *Bradyrhizobium*, *Subgroup_7*, *TRA3-20*, *Actinoplanes* and *Microvirga* (Fig. S1A).

**Table 3:** The relationship between soil dominant bacterial and fungal phyla and the soil characteristics.

	pH	TC	TN	C/N	TP	AN	AP	AK
Bacterial community								
Chao 1 index	−0.09	−0.40	−0.16	−0.36	−0.23	−0.24	−0.18	−0.31
Pielou_e index	**0.52** ^*^	0.25	**0.67** ^**^	**-0.76** ^**^	0.35	**0.59** ^*^	−0.16	0.45
Shannon index	0.23	−0.16	0.21	**−0.68** ^**^	−0.04	0.07	−0.21	0.01
Simpson index	0.34	−0.09	0.42	**−0.92** ^**^	0.12	0.33	−0.27	0.27
Fungal community								
Chao 1 index	0.47	0.45	0.39	−0.07	0.12	0.45	−0.21	0.44
Pielou_e index	0.36	0.54	0.35	0.08	0.19	0.50	−0.07	0.42
Shannon index	0.35	0.52	0.34	0.07	0.10	0.47	−0.14	0.37
Simpson index	0.33	0.50	0.30	0.11	0.26	0.43	0.03	0.42

**Notes.**

* Significant correlation at 0.05 level (two side).

** Significant correlation at 0.01 level (two side).

TC total carbon TN total nitrogen TP total phosphorus AN available nitrogen AP available phosphorus AK available potassium

For soil fungal community, Ascomycota, Basidiomycota, and Mortierellomycota were the predominant groups at the phylum level, accounting for 99.03%, 99.14%, 98.61% and 99.37% in PTAU, PTRP, PT, and PTVN, respectively (Fig. 2B). PTAU hold the highest Ascomycota with the relative abundance of 84.77%, and the lowest Basidiomycota with the relative abundance of 11.37%. In addition, lefse with LDA of 3.06 demonstrated that Ascomycota was the predominant group in PTAU, while, Basidiomycota was the predominant group in PTRP (Fig. 3B; Fig. S2B). At the genus level, the average relative abundance with more than 1% were *Knufia*, *Penicillium*, *Trechispora*, *Solicoccozyma*, *Tomentella*, *Gibberella*, *Talaromyces*, *Didymella*, *Mortierella*, *Cladophialophora*, *Geminibasidium*, *Fusarium*, *Sebacina*, and *Exophiala* (Fig. S1B).

**Figure 4:**
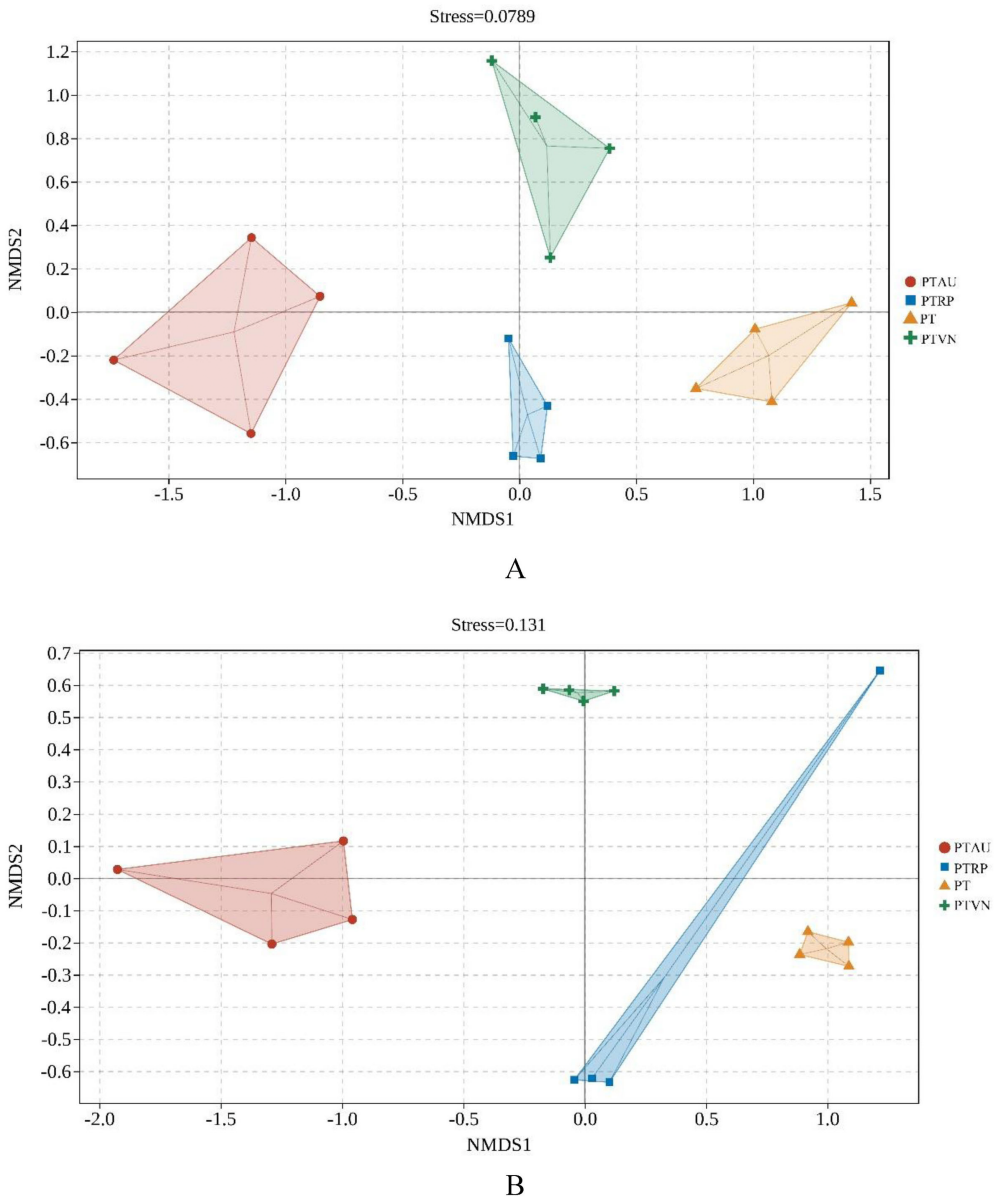
The NMDS of soil bacterial (A) and fungal (B) community structure based on Bray-cruits. PT, *Pinus tabuliformis*; PTAU, *Pinus tabuliformis*-*Armeniaca vulgaris* mixed forest; PTRP, *Pinus tabuliformis*-*Robinia pseudoacacia* mixed forest; PTVN, *Pinus tabuliformis*-*Vitex negundo* L. var. *heterophylla* mixed forest.

More interestingly, the NMDS based on Jaccard distance and the permanova demonstrated that soil bacterial community (stress = 0.08) (Fig. 4A; Table S2) and fungal community (stress = 0.13) (Fig. 4B; Table S3) among PT, PTAU, PTRP and PTVN showed clear difference. Soil microbial community in PTAU clearly separated from PTAR, PTVN, and PT along NMDS1, indicating that PTAU distinctly changed the soil microbial community structure (Fig. 4).

### The relationships between soil microbial community and soil characteristics

**Figure 5:**
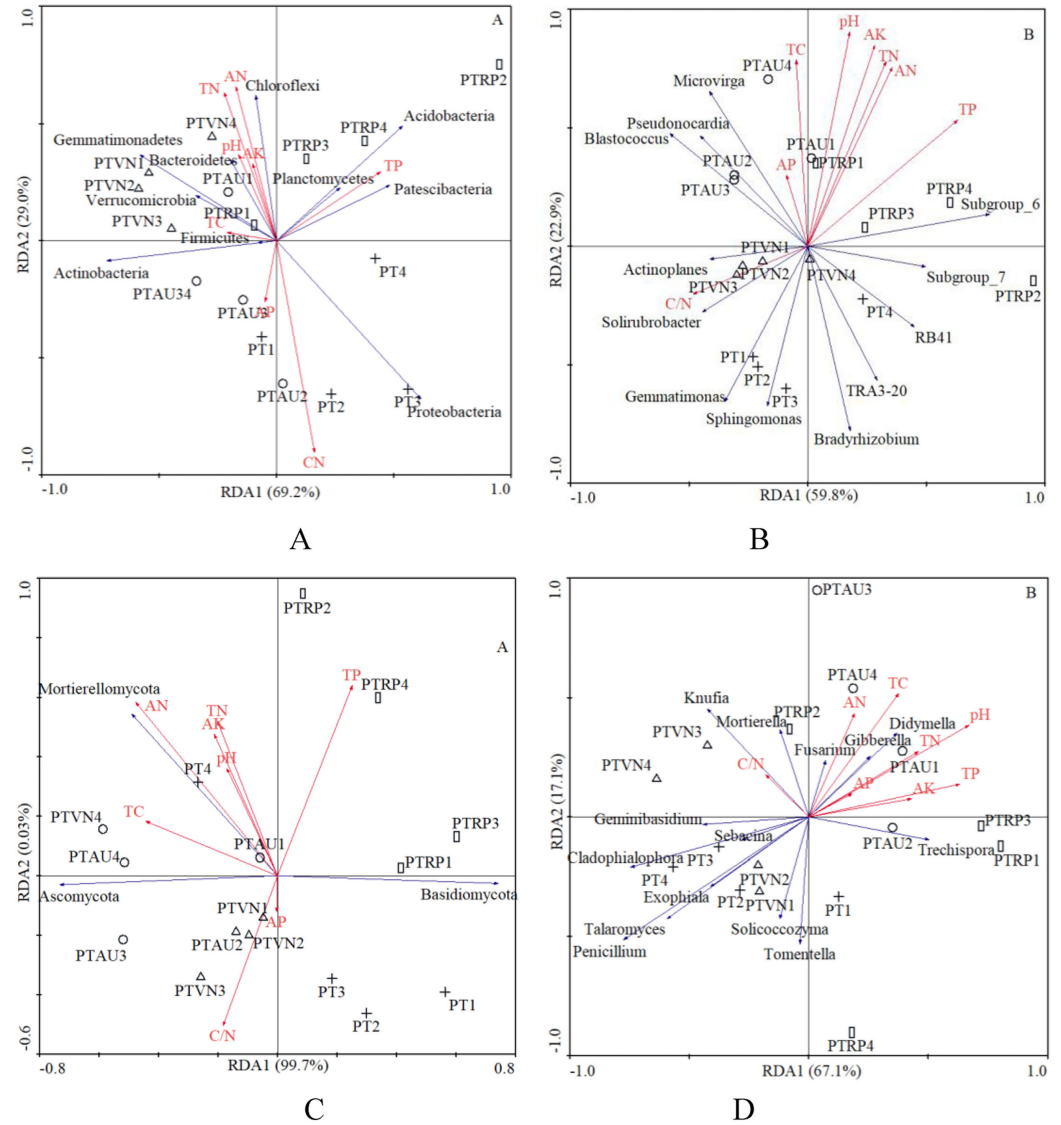
The RDA of soil characteristics on soil bacterial community at the phylum (A) and genus (B) level, and fungal community at the phylum (C) and genus (D) level. PT, *Pinus tabuliformis*; PTAU, *Pinus tabuliformis*-*Armeniaca vulgaris* mixed forest; PTRP, *Pinus tabuliformis*-*Robinia pseudoacacia* mixed forest; PTVN, *Pinus tabuliformis*-*Vitex negundo* L. var. *heterophylla* mixed forest. TC, total carbon; TN, total nitrogen; TP, total phosphorus; AN, available nitrogen; AP, available phosphorus; AK, available potassium.

In total, 8 chemical attributes, including pH, TC, TN, C/N, TP, AN, AP, and AK, were selected from all the physicochemical attributes measured in this study (Table 1). The RDA demonstrated that the first and second axis total explained 98.2% and 82.7% of the variance in the species environment relationship at the bacterial phylum and genus level, respectively, and 99.7% and 67.1% of the variance could be explained by the first and second axes at the fungal phylum and genus level, respectively, which indicated that these axes reflected the relationship between soil microbial communities and the environment to a large extent (Fig. 5).

In addition, Proteobacteria significantly declined with TN (*r* = −0.63, *P* < 0.01) and AN (*r* = −0.53, *P* < 0.05), while increased with soil C/N (*r* = 0.77, *P* < 0.01). Gemmatimonadetes existed negative relation with the TP (*r* = −0.62, *P* < 0.05). Chloroflexi appeared to be the significant positive correlation with soil pH (*r* = 0.62, *P* < 0.05), TN (*r* = 0.80, *P* < 0.01), TP (*r* = 0.50, *P* < 0.05), AN (*r* = 0.74, *P* < 0.01), and AK (*r* = 0.59, *P* < 0.05), while existed obviously negative relation with C/N (*r* = −0.60, *P* < 0.05). With regard to soil fungi, the relative abundance of Mortierellomycota increased with TN (*r* = 0.59, *P* < 0.05) and AN (*r* = 0.65, *P* < 0.01) (Table 4).

**Table 4:** The relationship between soil dominant bacterial and fungal phyla and the soil characteristics.

	pH	TC	TN	C/N	TP	AN	AP	AK
Proteobacteria	−0.41	−0.14	**−0.63** ^**^	**0.77** ^**^	0.06	**−0.53** ^*^	−0.08	−0.33
Actinobacteria	0.05	0.25	0.05	0.09	−0.29	0.02	0.06	0.01
Acidobacteria	0.00	−0.05	0.19	−0.26	0.32	0.19	−0.06	0.06
Bacteroidetes	0.45	−0.02	0.31	−0.30	0.05	0.28	−0.06	0.44
Gemmatimonadetes	−0.31	−0.47	−0.08	−0.48	**−0.62** ^*^	−0.07	−0.26	−0.40
Chloroflexi	**0.62** ^*^	0.50	**0.80** ^**^	**−0.60** ^*^	**0.50** ^*^	**0.74** ^**^	−0.01	**0.59** ^*^
Verrucomicrobia	−0.14	−0.50	−0.18	−0.47	−0.30	−0.28	−0.16	−0.36
Patescibacteria	−0.10	0.09	0.18	−0.02	0.39	0.21	0.24	0.09
Planctomycetes	0.07	0.16	0.19	−0.09	0.38	0.20	0.24	0.06
Firmicutes	−0.45	−0.09	−0.11	0.24	−0.36	0.04	0.14	−0.39
Ascomycota	0.23	0.42	0.25	0.19	−0.13	0.35	−0.09	0.26
Basidiomycota	−0.28	−0.40	−0.26	−0.14	0.08	−0.37	0.09	−0.30
Mortierellomycota	0.32	0.43	**0.59** ^*^	−0.26	0.21	**0.65** ^**^	−0.17	0.37

**Notes.**

* Significant correlation at 0.05 level (two side).

** Significant correlation at 0.01 level (two side).

TC total carbon TN total nitrogen TP total phosphorus AN available nitrogen AP available phosphorus AK available potassium

## Discussion

### Soil characteristics response to different vegetation types

In some areas, certain successes have been achieved in the near-natural transformation of artificial forests through selective logging and creating gaps (Luo et al., 2013). As anticipated, we observed that forest type conversion altered several of the measured soil attributes, including soil pH, TC, TN, C/N, TP, AN, and AK. What’s more, PTAU, PTVN, and PTRP could increase the soil pH, TC, TN, AN, and AK in different degrees, especially in PTAU. Previous study also showed that the soil pH, TC, TN, and TP concentrations in the *P. tabuliformis* forest were obviously lower than in a coniferous-broadleaved *P. tabuliformis* mixed forest (Chen et al., 2017). What’s more, the findings from Li et al. (2021a) demonstrated that soil pH in the monoculture *P. massoniana* forest plot existed abundantly lower than that in the broad-leaved tree species plantation. Furthermore, different aboveground vegetation types could affect the soil C/N ratio due to a change in litter composition and the microbial community structure were well documented (Zhang et al., 2013; Djukic et al., 2010). Our present study revealed that soil C/N in PT was significantly higher than in PTAU, PTVN, and PTRP, which was agreement with previous study (Monkai et al., 2018). All these findings demonstrated that trees can also change soil chemical parameters mostly *via* changes in litter quantity and quality and root exudates (Umemura & Takenaka, 2015).

### Soil microbial community diversity and composition response to different vegetation types

Our study revealed the impact of near-natural transformation on the diversity and composition of soil bacteria and fungi. Consistent with our hypothesis, near-natural forest transformation could improve soil bacterial Pielou_e index, and Simpson index, as well as soil fungal Chao1 index, which was consistent with previous study (Ding et al., 2021). In addition, we indicated that soil bacterial and fungal community composition among PT, PTAU, PTRP and PTVN showed clear variations, and PTAU visibly altered the soil microbial community structure, and our findings corroborated the previous reports (Sun et al., 2020; Jin et al., 2019; Liu et al., 2018). Collectively these findings nicely demonstrated that distinct difference of soil microbial communities is usually found to be associated with tree species (Meng et al., 2019).

In present study, Proteobacteria was the most abundant phylum in all soil samples under different vegetation types, and similar results have been repeatedly observed in past studies (Zhang & Lv, 2021; Deng et al., 2018; Zhao et al., 2012). While, Liu et al. (2013) reported that Acidobacteria was the predominant flora of soil bacterial communities, and the relative abundance of Acidobacteria ranked third in our study. Bacterioidetes, Gemmatimonadetes, Planctomycetes, Verrucomicrobia and Chloroflexi were also rich in all soil samples, which were basically consistent with previous studies on soil bacterial community in the Loess Plateau (Du et al., 2020; Yang, Wang & Shen, 2013). Among the dominant phyla, the relative abundance of Proteobacteria was the highest in PT, and PTVN hold the lowest relative abundance, which was in line with previous finding (Deng et al., 2018) indicated that the higher soil nutrients may be related to the higher relative abundance of Proteobacteria. Proteobacteria have been recognized as copiotrophic taxa (Davis, Sangwan & Janssen, 2010), which could be used as an indicator of soil nutrient status. In general, compared to oligotrophic taxa, copiotrophic taxa have higher N demands (Fontaine & Barot, 2005; Fierer, 2017). However, our reports documented that PT with lower nutrients N and a higher C/N ratio was beneficial to the survival of Proteobacteria. Actinobacteria, as important decomposers of cellulose and chitin (Pankratov, Dedysh & Zavarzin, 2006), is found to be abundant in diverse environments (Urich et al., 2008), which play a fundamental role in the turnover and circulation of organic matter. In our study, no significant difference of Actinobacteria were obtained among different vegetation types, while, findings from Li et al. (2021a) reported that the relative abundance of Actinobacteria in the mixed broadleaf-conifer forest presented lower than pure *P. massoniana* plantation. With regard to soil fungi, Ascomycota, Basidiomycota, and Mortierellomycota were the predominant groups at the phylum level (Fig. 2B), which appeared to be consistent with previous report documented that Ascomycota was the predominant fungal phylum (Deng et al., 2020). Basidiomycota, as one of the important components of functional group, participates in decomposing recalcitrant plant materials as well as plays an important role in biomass conversion and nutrient cycling (Morrison et al., 2016). Ascomycota, as a decomposer in soil, is a common fungal community in forest ecosystem and involves in many degradation processes (Riley et al., 2014), supporting the findings of earlier study (Li et al., 2021b).

Intriguingly, we found that *Subgroup_6*, *RB41*, and *Sphingomonas* were the dominant bacterial genera, as previously reported in the literature (Shi et al., 2017). *Sphingomonas,* as one of the abundant microbial groups in soil environment, played a crucial role in the determination of soil nutrient recycling. In addition, *Gemmatimonas* was a dominant genus in soil in our study, which had the ability of weathering minerals and solubilizing phosphate in various environments (Wei et al., 2019). Moreover, some bacterial genera, such as *Pseudonocardia*, *Biastococcus*, *RB41* and *Sphingomonas* were also notably different among different vegetation types. Previous investigations indicated that soil bacterial community were strongly influenced by different vegetation types (Mendes et al., 2015), which was confirmed again in our research.

### The relationship between soil characteristics and soil microbial communities

Soil characteristics, including soil TC, TN and TP, alter soil microbial community have been well documented (Zhao et al., 2019; Deng et al., 2018; Mendes et al., 2015). In present study, mixed coniferous and broadleaf forest increased soil bacterial Pielou_e index by increasing soil pH and TN (Table 3), as previous studies have shown that mixed broadleaf-conifer forest increased soil bacterial community diversity by increasing soil N, P and other nutrients in comparison to the monoculture *P. massoniana* (Li et al., 2021a). While, soil bacterial Pielou_e index, Shannon index, and Simpson index increased by decreasing soil C/N (Table 3). Nevertheless, no significant correlations of soil fungal community diversity with soil characteristics were observed (Table 3). All of these reports suggested that aboveground vegetation strongly influenced belowground microbial community diversity *via* soil chemical properties (Osborne et al., 2011).

In our study, the RDA results also showed that the soil microbial community composition were significantly correlated with soil characteristics (Fig. 5). Our results were in partial agreement with the findings of Yang et al. (2018), Rousk et al. (2010), and Li et al. (2021a) demonstrated that soil pH was a factor controlling the bacterial community structure. In the analysis of the relationships between soil chemical properties and the dominant bacterial communities, we found that Proteobacteria significantly declined with TN and AN, while increased with soil C/N. Gemmatimonadetes existed negative relation with the TP, however, previous study demonstrated that the relative abundance of Gemmatimonadetes was positively influenced by TC and SOM (Liu et al., 2020), suggesting that relatively high soil nutrients might stimulate the increase of the relative abundance of Gemmatimonadetes (Rosling et al., 2011). Chloroflexi appeared to be the significant positive correlation with soil pH, TN, TP, AN, and AK, similar with existed observation (Liu et al., 2020), while existed obviously negative relation with C/N, which was consistent with the investigations of the study from Wan et al. (2015). What’s more, other investigations also indicated that soil pH, TC and TN have impacts on the composition of bacterial communities (Cho, Kim & Lee, 2016; Barcenas-Moreno, Bååth & Rousk, 2016; Dawud et al., 2016). These findings collectively supported the idea that soil microorganisms were strongly linked with soil nutrients caused by different tree species (Siles & Margesin, 2016; Mueller et al., 2014; De Vries et al., 2012). Our research results revealed the influence of tree composition and soil nutrient availability on soil microbial diversity and composition, provided management guidance for incorporating soil microbial diversity and composition in plantation forest management.

## Conclusions

In summary, the data presented here showed evidence that the near-natural transformation of *P. tabuliformis* into *P. tabuliformis*-*Armeniaca vulgaris* resulted in significant improvements in soil pH, TC, TN, AN, and AK. Compared to PT, PTAU, PTRP and PTVN could increase soil bacterial Pielou_e index, and Simpson index, as well as soil fungal Chao1 index. What’s more, both soil bacterial and fungal community among PT, PTAU, PTRP and PTVN showed clear difference, and PTAU obviously altered the soil microbial community structure. Different vegetation type could change soil microbial community *via* altering soil characteristics. Our findings revealed the influence of tree composition and soil nutrient availability on soil microbial diversity and composition, and *P. tabuliformis-Armeniaca vulgaris* could better improve soil nutrients and thus increase soil microbial community diversity, which provided management guidance of the near-natural transformation of *P. tabuliformis* plantations.

##  Supplemental Information

10.7717/peerj.12098/supp-1 Supplemental Information 1 Soil bacterial and fungal community ranking at the top ten at the genus level with the relative abundance more than 1% PT: *Pinus tabuliformis*, PTAU: *Pinus tabuliformis*-*Armeniaca vulgaris* mixed forest, PTRP: *Pinus tabuliformis*-*Robinia pseudoacacia* mixed forest, PTVN: *Pinus tabuliformis*-*Vitex negundo* L. var. *heterophylla* mixed forest.

10.7717/peerj.12098/supp-2 Supplemental Information 2 LDA value distribution histogram PT: *Pinus tabuliformis*, PTAU: *Pinus tabuliformis*-*Armeniaca vulgaris* mixed forest, PTRP: *Pinus tabuliformis*-*Robinia pseudoacacia* mixed forest, PTVN: *Pinus tabuliformis*-*Vitex negundo* L. var. *heterophylla* mixed forest.

10.7717/peerj.12098/supp-3 Supplemental Information 3 Basic information of sampling plots PT: *Pinus tabuliformis*, PTAU: *Pinus tabuliformis*-*Armeniaca vulgaris* mixed forest, PTRP: *Pinus tabuliformis*-*Robinia pseudoacacia* mixed forest, PTVN: *Pinus tabuliformis*-*Vitex negundo* L. var. *heterophylla* mixed forest.

10.7717/peerj.12098/supp-4 Supplemental Information 4 Individual measurements for Table 1 TC: total carbon, TN: total nitrogen, TP: total phosphorus, AN: available nitrogen, AP: available phosphorus, AK: available potassium, PT: *Pinus tabuliformis*, PTAU: *Pinus tabuliformis*-*Armeniaca vulgaris* mixed forest, PTRP: *Pinus tabuliformis*-*Robinia pseudoacacia* mixed forest, PTVN: *Pinus tabuliformis*-*Vitex negundo* L. var. *heterophylla* mixed forest. Different uppercase letters in the same row indicate significant difference at the 0.01 level, and different lowercase letters in the same row indicate significant differences at the 0.05 level.

## Additional Information and Declarations

### Data Availability

The following information was supplied regarding data availability: The high-throughput sequencing raw data of bacteria and fungi are available at NCBI: PRJNA720506.
