# Supplementary material for: Near-natural transformation of Pinus tabuliformis better improve soil nutrients and soil microbial community
Source: PeerJ. 2021 Sep 23;9:e12098. doi: 10.7717/peerj.12098 (PMC8465996; doi:10.7717/peerj.12098)
Supplement: Supplemental Information 2 — PT: Pinus tabuliformis, PTAU: Pinus tabuliformis-Armeniaca vulgaris mixed forest, PTRP: Pinus tabuliformis-Robinia pseudoacacia mixed forest, PTVN: Pinus tabuliformis-Vitex negundo L. var. heterophylla mixed forest. [file peerj-09-12098-s002.docx]

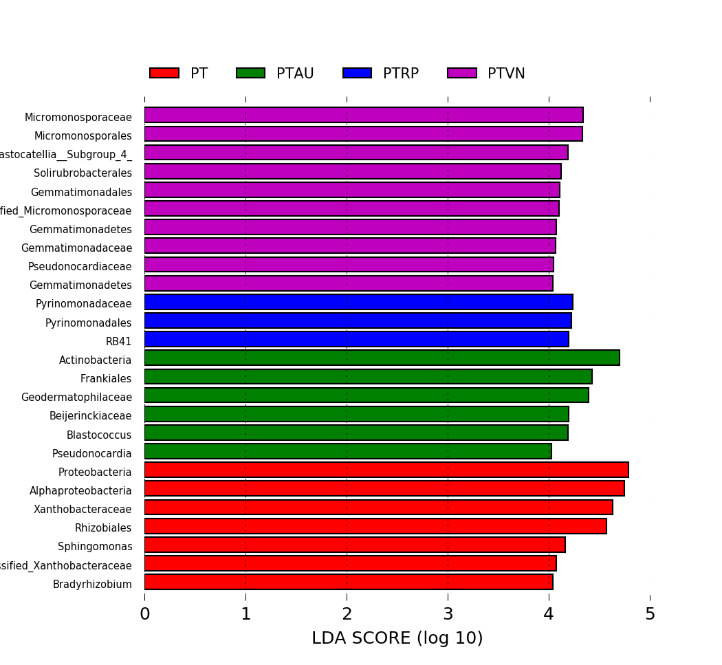


A


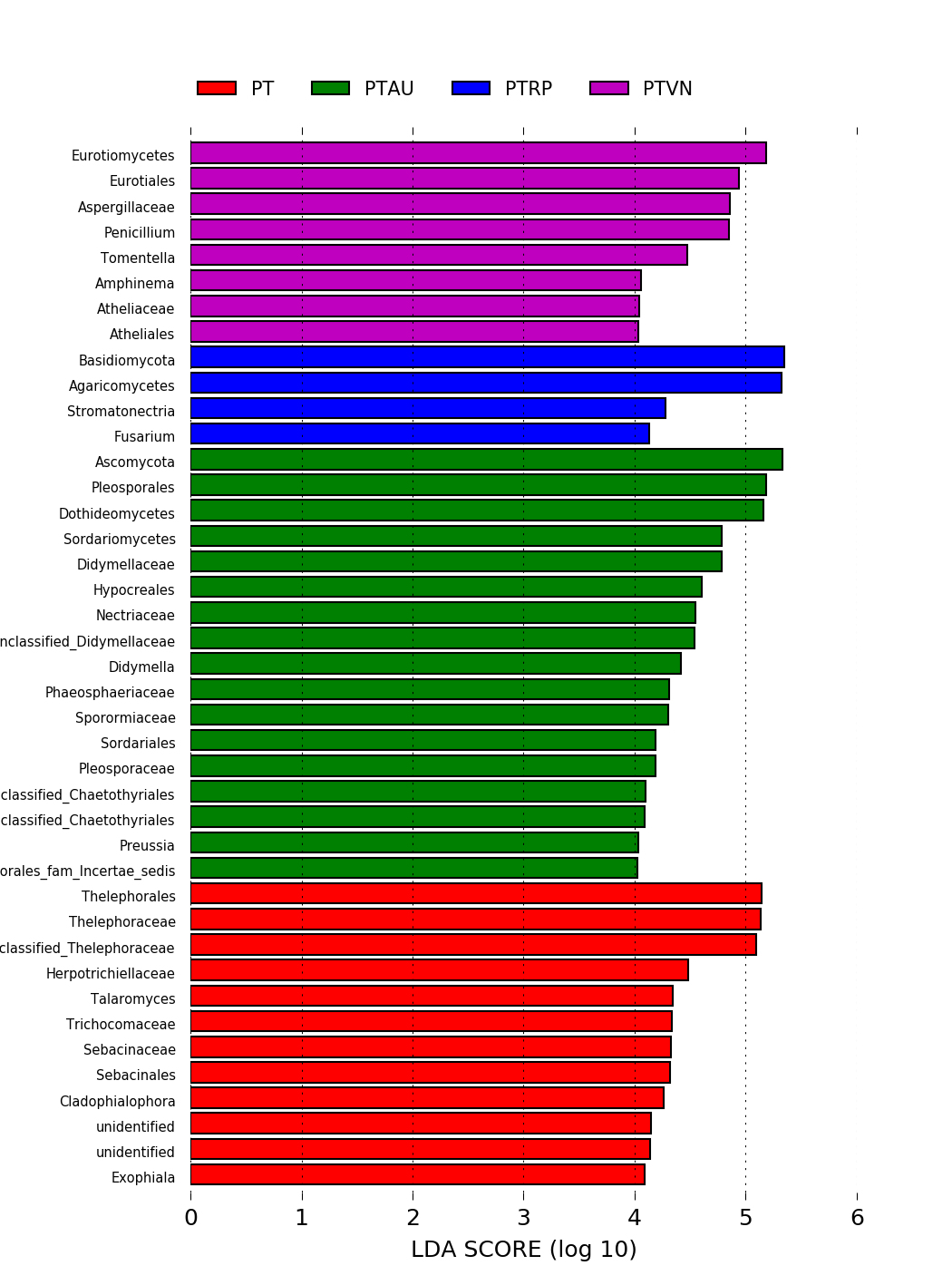


B

Figure S2 LDA value distribution histogram. PT: *Pinus tabuliformis*, PTAU: *Pinus* *tabuliformis*-*Armeniaca* *vulgaris* mixed forest, PTRP: *Pinus* *tabuliformis*-*Robinia* *pseudoacacia* mixed forest, PTVN: *Pinus* *tabuliformis*-*Vitex* *negundo* L. var. *heterophylla* mixed forest.
