# Supplementary material for: Near-natural transformation of Pinus tabuliformis better improve soil nutrients and soil microbial community
Source: PeerJ. 2021 Sep 23;9:e12098. doi: 10.7717/peerj.12098 (PMC8465996; doi:10.7717/peerj.12098)
Supplement: Supplemental Information 3 — PT: Pinus tabuliformis, PTAU: Pinus tabuliformis-Armeniaca vulgaris mixed forest, PTRP: Pinus tabuliformis-Robinia pseudoacacia mixed forest, PTVN: Pinus tabuliformis-Vitex negundo L. var. heterophylla mixed forest. [file peerj-09-12098-s003.docx]

Table S1

Basic information of sampling plots

| Stand type | Age | Elevation | Slope | Canopy density | DBH of *Pinus tabuliformis* | Height of *Pinus tabuliformis* |
| --- | --- | --- | --- | --- | --- | --- |
| PTAU | 54 | 720m | 10° | 0.50 | 17.2cm | 16.1m |
| PTRP | 54 | 700m | 10° | 0.20 | 16.7cm | 15.3m |
| PT | 54 | 700m | 10° | 0.67 | 15.5cm | 14.6m |
| PTVN | 54 | 730m | 3° | 0.67 | 16.3cm | 14.8m |

Note:

PT: *Pinus tabuliformis*, PTAU: *Pinus* *tabuliformis*-*Armeniaca* *vulgaris* mixed forest, PTRP: *Pinus* *tabuliformis*-*Robinia* *pseudoacacia* mixed forest, PTVN: *Pinus* *tabuliformis*-*Vitex* *negundo* L. var. *heterophylla* mixed forest.
